# Comparative effectiveness of TAP block, EOIP block, and standard care for postoperative analgesia in renal transplantation: a retrospective study

**DOI:** 10.1016/j.bjane.2025.844692

**Published:** 2025-10-24

**Authors:** Karam Azem, Roussana Aranbitski, Safo Awad, Omer Shpack, Alexander Peres, Rotem Argov, Omer Kaplan, Anatoliy Artyukh, Andrey Khinchuk, Michael Yohay Stav, Philip Heesen, Sharon Orbach-Zinger, Eviatar Nesher, Shai Fein

**Affiliations:** a Tel Aviv University, Rabin Medical Centre and the Faculty of Medical and Health Sciences, Beilinson Hospital, Department of Anesthesiology, Tel Aviv, Israel; b University of Strathclyde, Department of Mathematics and Statistics, Glasgow, Scotland; c Faculty of Medicine, University of Zurich, Zurich, Switzerland; d Tel Aviv University, Rabin Medical Centre and the Faculty of Medical and Health Sciences, Beilinson Hospital, Department of Organ Transplantation, Tel Aviv, Israel

**Keywords:** Analgesics, opioid, Kidney transplantation, Living donors, Nerve block, Pain management, Pain, postoperative

## Abstract

**Background:**

Effective pain management following renal transplantation is crucial. While various regional analgesic techniques have been studied, the optimal approach remains unclear. We compared the additive value of Transversus Abdominis Plane (TAP) and External Oblique Intercostal Plane (EOIP) blocks to Standard Care (SC) on postoperative pain and opioid consumption.

**Methods:**

This retrospective study included 237 renal transplant recipients (127 SC, 75 TAP, 35 EOIP) between January 2023 and December 2024. Multivariable regression analysis assessed the association of block type on postoperative pain and opioid consumption.

**Results:**

TAP block was associated with significantly lower pain scores than SC during the first eight postoperative hours (5.0 vs. 7.0, *p* < 0.001). Pre-incision TAP block demonstrated the most significant reduction in both pain scores (β = -2.21, 95% CI -3.38 to -1.05, *p* < 0.001) and opioid consumption (β = -13.56, 95% CI: -21.59 to -5.52, *p* = 0.001). EOIP block showed no significant advantages over SC and was associated with higher opioid consumption compared to TAP block.

**Conclusion:**

Pain predominantly manifested in the first eight postoperative hours. TAP block, particularly when administered pre-incision, was associated with superior pain control compared to SC or EOIP block. Living donor recipients experienced significantly higher pain scores regardless of technique, warranting further investigation.

## Introduction

Though Renal Transplantation (RT) is the definitive treatment for end-stage renal disease, offering significant improvements in quality of life and survival compared to dialysis,^1^ it is frequently associated with moderate to severe postoperative pain.^2^ Pain management in this specific patient population is particularly challenging due to impaired renal function, which restricts the use of many analgesics.^3^

Enhanced Recovery After Surgery (ERAS) protocols have emerged as a promising approach for RT, reducing both pain scores and opioid consumption while leading to shorter hospital stays, decreased morbidity, and improved patient satisfaction.^4^, ^5^, ^6^

As part of the ERAS pathway for RT, various regional analgesic techniques have been investigated.^7^, ^8^, ^9^, ^10^, ^11^, ^12^ The Transversus Abdominis Plane (TAP) block has emerged as one of the most widely studied regional techniques in this context. However, despite promising results, there is still no consensus on the optimal regional analgesia approach for this surgery.

The External Oblique Intercostal Plane (EOIP) block has recently emerged as a promising novel technique, gaining attention for both its simplicity and effectiveness.^13^ By blocking the intercostal nerves from T6 to T11, it provides effective analgesia for the anterolateral upper abdominal wall.^13^ However, the Gibson incision used for renal transplantation typically involves dermatomes from T10/11 to L1/2, which extends beyond the EOIP block coverage. Although the EOIP block only partially covers the dermatomal distribution of the Gibson incision, its potential utility in RT has been suggested in the literature. Notably, in the original case series by Elsharkawy et al. that introduced this block, one of the 22 reported patients had received the EOIP block for RT.^13^ The block's practical advantages ‒ including technical simplicity, distance from the surgical site, and elimination of dressing manipulation ‒ make it an attractive option for investigation in this setting.

Given the absence of consensus guidelines for optimal analgesic protocol in RT and the theoretical potential of EOIP block despite partial dermatomal coverage, we conducted this retrospective study to compare the efficacy of commonly used analgesic techniques and identify factors associated with improved pain control and reduced opioid consumption.

## Methods

### Ethics, design, and settings

This retrospective exploratory study was conducted at Rabin Medical Center, Beilinson Hospital, Israel (the Israeli National Transplantation Center). Ethical approval (0649–24-RMC) was provided by the Institutional Review Board (Chairperson Prof. Ran Tur-Kaspa) in January 2025. Written informed consent was waived due to the retrospective, non-interventional nature of the study. This manuscript adheres to the STROBE statement.

### Study population

We included patients aged 18 years, and above who underwent RT and had complete medical records available. Patients were excluded if they had undergone dual organ transplantation (such as liver, and RT or pancreas and RT), experienced intraoperative bleeding requiring transfusion of more than three blood products, had surgeries lasting longer than six hours, received additional regional analgesic techniques beyond the study focus (such as quadratus lumborum, erector spinae plane, intercostal, ilioinguinal-iliohypogastric blocks, or combination of more than one block), required rescue blocks in the Post Anesthesia Care Unit (PACU), were transferred to PACU while on mechanical ventilation, had a PACU stay exceeding eight hours, or were directly transferred to the Intensive Care Unit (ICU) immediately after surgery.

### Anesthetic and analgesic care

At the study institution, RT recipients typically receive the following Standard Care (SC), although minor variations in drug selection and dosing may occur based on the anesthesiologist's clinical judgment and patient-specific conditions.

## Intraoperative care

Anesthesia was induced intravenously using fentanyl (1‒3 mg.kg^-1^), propofol (1‒2 mg.kg^-1^), and either rocuronium (0.6‒1.2 mg.kg^-1^) or atracurium (0.3‒0.5 mg.kg^-1^). Dosing was individualized based on patient characteristics and comorbidities. Anesthesia was maintained using volatile anesthetic agents. Unless contraindicated, patients received intraoperative multimodal analgesia consisting of intravenous paracetamol (1 g), tramadol (100 mg), and dipyrone (1 g).

## Regional analgesic techniques

Based on anesthesiologist discretion, in addition to the SC, some patients received ultrasound-guided (GE Healthcare, Venue GO, Chicago, IL, USA) TAP or EOIP block, either pre- or post-incision (at the end of surgery). For the TAP block, a high-frequency linear probe (6‒12 MHz) was used for imaging, and a 22 *G* × 50 mm or 22 *G* × 80 mm needle (SonoTAP®; PAJUNK® GmbH, Medizintechnologie, Geisingen, Germany) was used to inject local anesthetic into the plane between the internal oblique and transversus abdominis muscles at the triangle of Petit.^14^ For the EOIP block, a high-frequency linear probe (6‒12 MHz) was used for imaging, and a 22 *G* × 50 mm needle (SonoTAP®; PAJUNK® GmbH) was used to inject a local anesthetic into the fascial plane between the external and internal oblique muscles at the level of the 6th to 7th ribs along the anterior axillary line.^13^ The volume of the local anesthetic was adjusted according to the patient's weight while maintaining local anesthetic safety limits. Typically, bupivacaine 0.25 % with epinephrine (Bupicain® with epinephrine, Monico spa, Venezia, Italy) was injected at a dose of 0.3‒0.6 mL.kg^-1^.

## Postoperative care

In the PACU, breakthrough pain was managed with intravenous tramadol 100 mg, followed by titrated doses of intravenous morphine (3‒5 mg) as needed. All patients received scheduled intravenous paracetamol (1 g) and dipyrone (1 g) every eight hours. After transferring to the surgical ward, patients continued with scheduled paracetamol and dipyrone. For breakthrough pain (NRS > 5), patients received either tramadol (100 mg), oxycodone (5‒10 mg), or combination analgesics such as paracetamol 325 mg/oxycodone 7.5 mg or paracetamol 500 mg/caffeine 30 mg/codeine phosphate 10‒15 mg.

### Study groups

Patients were categorized into three groups based on the analgesic technique received: SC group, which received SC alone; TAP group, which received SC and TAP block; EOIP group, which received SC and EOIP block.

### Study objectives

The objectives of this retrospective study were to:

1. Compare pain scores and opioid consumption during the first 72 hours postoperatively among the study groups and identify factors associated with these outcomes;
2. Compare the incidence of postoperative complications, PACU length of stay, and hospital length of stay among the study groups;
3. Assess the impact of regional block timing (pre-incision vs. post-incision) on analgesic efficacy in the TAP and EOIP groups.

### Measurements and data collection

Data were extracted from the electronic medical record systems (Metavision, iMDSoft, Israel; and Chameleon™, Elad Health, Israel). Pain scores were assessed using the numeric rating scale (NRS), recorded at least twice per 8-hour shift per institutional protocol, with additional measurements for patients reporting pain. The first 24 hours were divided into 8-hour intervals (0‒8, 8‒16, 16‒24 hours) to provide higher temporal resolution during the period of expected peak postoperative pain, followed by 24‒48 and 48‒72 hour periods. The maximum NRS value from all recordings within each time period was used to capture clinically significant pain episodes and prevent underestimation in patients with intermittent severe pain. Opioid consumption was quantified as the oral Morphine Milligram Equivalents (MME) and calculated both for intraoperative and each postoperative period. Oral MME was calculated using standardized conversion factors (e.g., 0.2 for 1 mg of intravenous tramadol, 3 for 1 mg of intravenous morphine, 1.5 for 1 mg of oral oxycodone, 0.15 for 1 mg of oral codeine, and 300 for 1 mg of intravenous fentanyl).^15^

In addition, sociodemographic and medical history data were collected, including age, gender, Body Mass Index (BMI), the American Society of Anesthesiologists (ASA) physical status, comorbidities, and concurrent medications. Intraoperative data included surgery duration and type (living or deceased donor), analgesic technique (SC, TAP block, or EOIP block), and block timing. Postoperative data included analgesic and anesthetic drug administration up to 72 hours postoperatively, complications (such as reoperation, surgical site infection, and unplanned ICU admission) within 72 hours, PACU and hospital length of stay, in-hospital mortality, and 30-day mortality.

### Statistical methods

Descriptive statistics were used to summarize the data. The distribution of continuous variables was assessed visually using histograms and Q-Q plots. As none of the continuous variables were normally distributed, continuous variables were reported as medians with interquartile ranges [25th, 75th percentiles] and compared using the Kruskal-Wallis test, followed by Dunn’s post hoc test with Bonferroni correction for pairwise comparisons. Categorical variables were presented as counts and percentages ( %) and compared using the Chi-Square test or Fisher’s exact test, as appropriate.

Multivariable regression models were employed to evaluate the association between analgesic techniques and pain scores and opioid consumption, adjusted for potential confounders, including age, gender, BMI, diabetes mellitus, analgesic modality, and intraoperative MME. Regression results were reported as beta coefficients with corresponding 95 % Confidence Intervals (95 % CIs) and p-values. Subgroup analyses were performed to investigate the impact of TAP block timing (pre- vs. post-incision) on pain scores and opioid consumption. All statistical tests were two-sided; a p-value < 0.05 was considered statistically significant. Statistical analyses were conducted using *R* statistical software (version 4.4.1).

## Results

**Figure 1:**
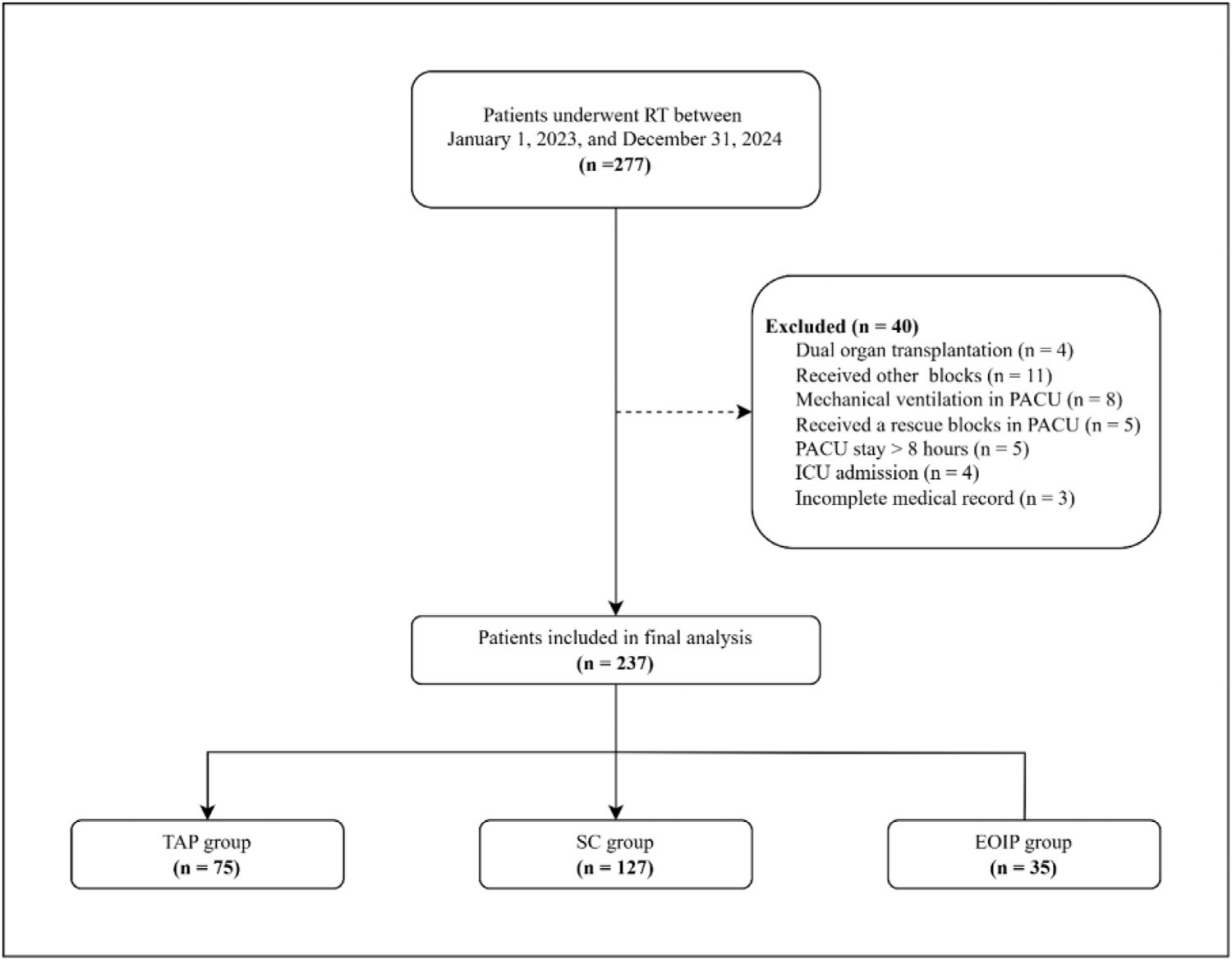
Patient inclusion flow diagram. EOIP, External Oblique Intercostal Plane; ICU, Intensive Care Unit; PACU, Post Anesthesia Care Unit; RT, Renal Transplantation; SC, Standard Care; TAP, Transversus Abdominis Plane.

Between January 1, 2023, and December 31, 2024, a total of 237 patients met the study inclusion criteria and were included in the analysis. Of these, 127 were in the SC group, 75 in the TAP group, and 35 in the EOIP group. The participant inclusion flow diagram is illustrated in Figure 1.

Table 1 presents detailed baseline characteristics and intraoperative data stratified by analgesic technique. Baseline characteristics were comparable across the groups, with a median age of 55.0 (43.0, 64.0) years and a male predominance of 168 (71 %). There were no significant differences in BMI, ASA physical status, or key comorbidities. Most kidney grafts, 165 (70 %), were from living-related donors. Of the regional blocks performed, 81 (74 %) were administered post-incision. The median duration of surgery was 3.4 (3.0, 3.9) hours. Intraoperative opioid consumption, measured in MME, differed significantly among groups, with the SC group requiring higher doses compared to the TAP group (95.0 [80.0, 110.0] vs. 80.0 [80.0, 95.0], *p* = 0.011).

**Table 1:** Baseline patient characteristics and intraoperative data by analgesic technique.

	Overall (*n* = 237)	SC (*n* = 127)	TAP block (*n* = 75)	EOIP block (*n* = 35)	Overall	Post hoc
					*p*-value	*p*-value
**Baseline patient characteristics**						
Median age [IQR], years	55.0 [43.0, 64.0]	58.0 [44.5, 64.5]	55.0 [42.5, 64.0]	49.0 [36.0, 60.5]	0.107	
**Sex, n ( %)**					0.507	
Male	168 (71 %)	94 (74 %)	51 (68 %)	23 (66 %)		
Female	69 (29 %)	33 (26 %)	24 (32 %)	12 (34 %)		
Median BMI [IQR], kg.m^-2^	26.0 [22.9, 29.4]	26.2 [23.3, 30.0]	25.4 [21.9, 28.7]	25.9 [23.1, 29.0]	0.3	
**ASA physical status**					0.118	
Class III	154 (65 %)	80 (63 %)	55 (73 %)	19 (54 %)		
Class IV	83 (35 %)	47 (37 %)	20 (27 %)	16 (46 %)		
**Background disease, n ( %)**						
Hypertension	146 (62 %)	83 (65 %)	44 (59 %)	19 (54 %)	0.402	
Ischemic heart disease	50 (21 %)	32 (25 %)	14 (19 %)	4 (11 %)	0.173	
Congestive heart failure	15 (6.3 %)	10 (7.9 %)	3 (4.0 %)	2 (5.7 %)	0.572	
Peripheral vascular disease	14 (5.9 %)	6 (4.7 %)	6 (8.0 %)	2 (5.7 %)	0.598	
Atrial fibrillation	7 (3.0 %)	2 (1.6 %)	4 (5.3 %)	1 (2.9 %)	0.256	
OSA	8 (3.4 %)	3 (2.4 %)	3 (4.0 %)	2 (5.7 %)	0.427	
Obesity	54 (23 %)	32 (25 %)	14 (19 %)	8 (23 %)	0.565	
COPD/Asthma	12 (5.1 %)	9 (7.1 %)	1 (1.3 %)	2 (5.7 %)	0.182	
Active smoking	48 (20 %)	24 (19 %)	17 (23 %)	7 (20 %)	0.812	
CVA/TIA	14 (5.9 %)	7 (5.5 %)	4 (5.3 %)	3 (8.6 %)	0.696	
Diabetes mellitus	61 (26 %)	33 (26 %)	18 (24 %)	10 (29 %)	0.874	
**Intraoperative data**						
Donation type, n ( %)					0.180	
Deceased donor	72 (30 %)	42 (33 %)	24 (32 %)	6 (17 %)		
Living donor	165 (70 %)	85 (67 %)	51 (68 %)	29 (83 %)		
Block timing					0.167	
Before incision	29 (26 %)		23 (31 %)	6 (17 %)		
After incision	81 (74 %)		52 (69 %)	29 (83 %)		
**Intraoperative MME, median [IQR]**	80.0 [80.0–110.0]	95.0 [80.0–110.0]	80.0 [80.0–95.0]	80.0 [80.0–92.5]	**0.004**	**0.011**^a^
**Surgery duration, median [IQR], hours**	3.4 [3.0, 3.9]	3.3 [2.9, 3.8]	3.5 [3.0, 3.9]	3.5 [3.1, 3.9]	0.314	

Continuous variables were reported as medians with interquartile ranges (25th and 75th percentiles). Categorical variables were presented as counts and percentages ( %). Statistical comparisons across groups were performed using the Kruskal-Wallis test for continuous variables and Chi-Square or Fisher's exact test for categorical variables. Adjusted pairwise comparisons were conducted using Dunn’s test with Bonferroni correction for significant KW tests.

a Significant difference between SC and TAP. BMI, Body Mass Index; COPD, Chronic Obstructive Pulmonary Disease; CVA, Cerebrovascular Accident; EOIP, External Oblique Intercostal Plane; ICU, Intensive Care Unit; IQR, Interquartile Range; KW, Kruskal-Wallis; MME, Morphine Milligram Equivalent; OSA, Obstructive Sleep Apnea; PACU, Post Anesthesia Care Unit; SC, Standard Care; TIA, Transient Ischemic Attack; TAP, Transversus Abdominis Plane.

**Figure 2:**
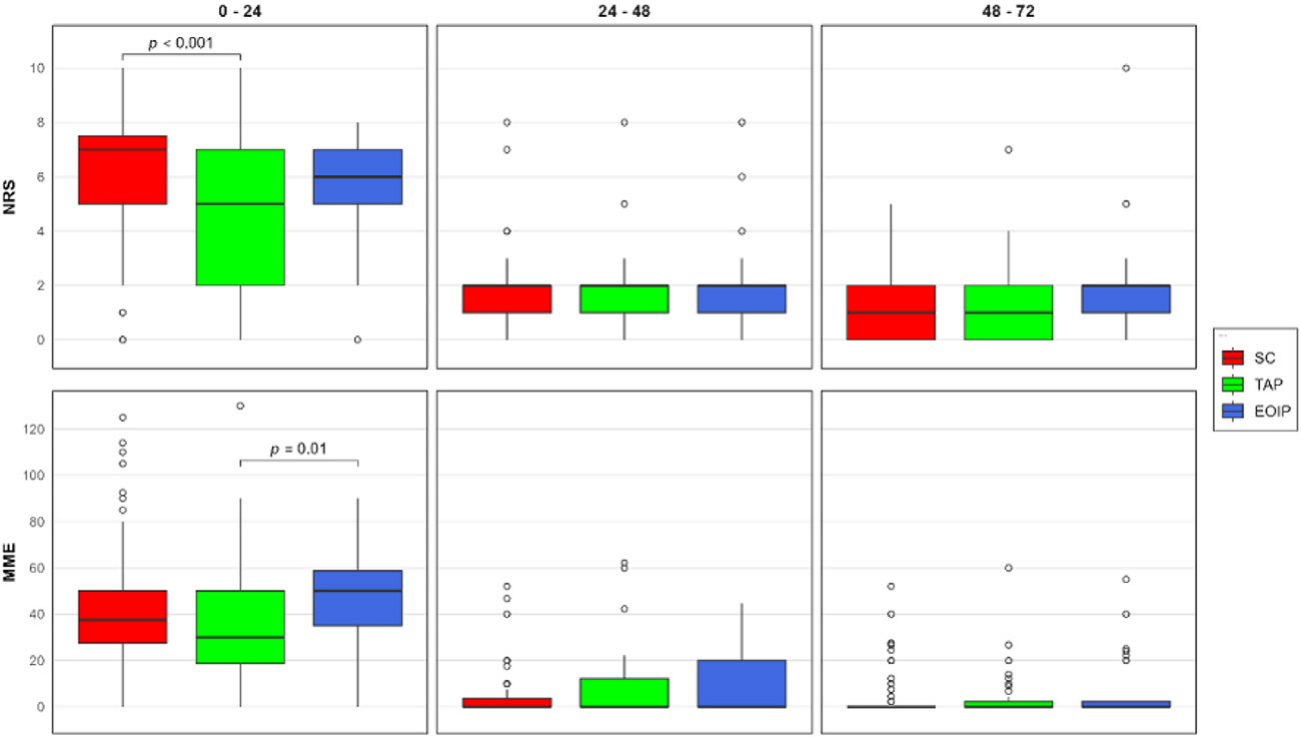
Pain scores and opioid consumption by analgesic technique during the first 72 postoperative hours. The upper panel represents pain (maximum NRS pain score) measured over time. The lower panel represents opioid consumption (MME) over time. In the box plots, medians are indicated by the central lines inside the boxes, IQRs are represented by the boxes, and the whiskers extend to 1.5 times the IQRs. Outliers, denoted by circles, are also displayed to highlight extreme data points. Significant differences between groups are shown with brackets and *p*-values from post-hoc Dunn's test with Bonferroni correction. EOIP, External Oblique Intercostal Plane; IQR, Interquartile Range; MME, Morphine Milligram Equivalent; NRS, Numeric Rating Scale; SC, Standard Care; TAP, Transversus Abdominis Plane.

Postoperative outcomes are detailed in Table 2. Maximum pain scores in the first 24 hours were significantly different across groups (*p* < 0.001), with the TAP block group showing lower scores compared to the SC group (5.0 [2.0, 7.0] vs. 7.0 [5.0, 7.5], *p* < 0.001). This difference was primarily driven by pain scores in the first eight hours postoperatively (5.0 [1.5, 7.0] vs. 7.0 [5.0, 7.5], *p* < 0.001). Beyond eight hours, pain scores were comparable across all groups, with median NRS scores remaining below two from eight to 72 hours postoperatively. Total opioid consumption in the first 24 hours differed significantly between groups (*p* = 0.009), with higher consumption in the EOIP group compared to the TAP group (50.0 [35.0–58.8] vs. 30.0 [18.6, 50.0] MME, *p* = 0.010). This difference was most pronounced in the first eight hours after surgery (50.0 [30.0, 50.0] vs. 30.0 [15.0, 50.0] MME, *p* = 0.015). Beyond 24 hours, opioid requirements were minimal across all groups, with a median consumption of 0 MME and no significant differences between groups. Figure 2 illustrates the temporal changes in pain scores and opioid consumption across the three groups during the first 72 postoperative hours.

**Table 2:** Postoperative outcomes by analgesic technique.

	Overall (*n* = 237)	SC (*n* = 127)	TAP block (*n* = 75)	EOIP block (*n* = 35)	Overall	Post hoc
					*p*-value	*p*-value
**Pain scores, median [IQR]**						
Maximum NRS for 0‒24 hours	6.0 [4.0–7.0]	7.0 [5.0–7.5]	5.0 [2.0–7.0]	6.0 [5.0–7.0]	**< 0.001**	**< 0.001**^a^
0‒8 hours	6.0 [4.0–7.0]	7.0 [5.0–7.5]	5.0 [1.5–7.0]	6.0 [5.0–7.0]	**< 0.001**	**< 0.001**^a^
8‒16 hours	1.0 [0.0–1.0]	1.0 [0.0–1.0]	1.0 [0.0–1.5]	1.0 [0.0–1.0]	0.426	
16‒24 hours	1.0 [0.0–2.0]	1.0 [0.0–2.0]	1.0 [0.0–2.0]	1.0 [0.0–2.0]	0.987	
Maximum NRS for 24‒48 hours	2.0 [1.0–2.0]	2.0 [1.0–2.0]	2.0 [1.0–2.0]	2.0 [1.0–2.0]	0.692	
Maximum NRS for 48‒72 hours	1.0 [0.0–2.0]	1.0 [0.0–2.0]	1.0 [0.0–2.0]	2.0 [1.0–2.0]	0.077	
**Opioid consumption, median [IQR]**						
Total MME for 0‒24 hours	35.0 [20.0–50.0]	37.5 [27.5–50.0]	30.0 [18.6–50.0]	50.0 [35.0–58.8]	**0.009**	**0.010**^b^
0‒8 hours	30.0 [20.0–50.0]	30.0 [20.0, 50.0]	30.0 [15.0, 50.0]	50.0 [30.0, 50.0]	**0.012**	**0.015**^b^
8‒16 hours	0.0 [0.0–0.0]	0.0 [0.0–0.0]	0.0 [0.0–0.0]	0.0 [0.0–0.0]	0.408	
16‒24 hours	0.0 [0.0–0.0]	0.0 [0.0–0.0]	0.0 [0.0–0.0]	0.0 [0.0–1.1]	0.882	
Total MME for 24‒48 hours	0.0 [0.0–9.8]	0.0 [0.0–3.8]	0.0 [0.0–12.3]	0.0 [0.0–20.0]	0.316	
Total MME for 48‒72 hours	0.0 [0.0–2.3]	0.0 [0.0–0.0]	0.0 [0.0–2.3]	0.0 [0.0–2.3]	0.394	
**Postoperative complications, n ( %)**						
ICU admission	6 (2.5 %)	4 (3.1 %)	1 (1.3 %)	1 (2.9 %)	0.736	
Surgical site infection	7 (3.0 %)	6 (4.7 %)	0 (0 %)	1 (2.9 %)	0.169	
Reoperation	5 (2.1 %)	2 (1.6 %)	2 (2.7 %)	1 (2.9 %)	0.699	
**PACU stay, median [IQR], hours**	3.3 [2.7, 4.1]	3.3 [2.8, 4.1]	3.2 [2.5, 3.8]	3.0 [2.6, 4.3]	0.282	
**Hospital stay, median [IQR], days**	6.5 [6.3, 8.5]	6.7 [6.3, 9.4]	6.7 [6.3, 8.3]	6.3 [6.0, 7.3]	0.108	

Continuous variables were reported as medians with interquartile ranges (25th and 75th percentiles). Statistical comparisons across groups were performed using the Kruskal-Wallis test for continuous variables and Chi-Square or Fisher's exact test for categorical variables. Adjusted pairwise comparisons were conducted using Dunn’s test with Bonferroni correction for significant KW tests:

a Significant difference between SC and TAP.

b Significant difference between TAP and EOIP. EOIP, External Oblique Pentercostal Plane; ICU, Intensive Care Unit; IQR, Interquartile Range; KW, Kruskal-Wallis; MME, Morphine Milligram Equivalent; NRS, Numerical Rating Scale; PACU, Post Anesthesia Care Unit; SC, Standard Care; TAP, Transversus Abdominis Plane.

Multivariable regression analysis adjusting for age, sex, BMI, diabetes mellitus, living donor status, and intraoperative MME confirmed the independent association between TAP block and early postoperative pain control (Table 3). Compared to SC, TAP block was associated with significantly lower pain scores (β = −1.60, 95 % CI: −2.36 to −0.84, *p* < 0.001) and reduced opioid consumption (β = −7.32, 95 % CI: −12.56 to −2.08, *p* = 0.006) in the first eight hours after surgery. EOIP block showed no significant difference from SC in either pain scores (β = −0.55, 95 % CI: −1.55 to 0.45, *p* = 0.282) or opioid consumption (β = 1.22, 95 % CI: −5.69 to 8.13, *p* = 0.728). Living donor transplantation was independently associated with higher pain scores (β = 0.91, 95 % CI: 0.13 to 1.68, *p* = 0.022).

**Table 3:** Multivariable linear regression analysis of factors associated with pain scores and opioid consumption during the first eight postoperative hours.

	NRS 0h – 8h		MME 0h – 8h	
	β coefficient (95 % CI)	*p*-value	β coefficient (95 % CI)	*p*-value
**Age, years**	0.00 (−0.03, 0.02)	0.926	−0.12 (−0.30, 0.06)	0.176
**Male sex**	−0.13 (−0.87, 0.60)	0.720	−0.90 (−6.00, 4.19)	0.727
**BMI, kg.m^-2^**	−0.05 (−0.12, 0.02)	0.140	−0.36 (−0.85, 0.13)	0.146
**Diabetes mellitus**	−0.39 (−1.23, 0.44)	0.354	−1.69 (−7.44, 4.07)	0.564
**Living donor**	0.91 (0.13, 1.68)	**0.022**	4.97 (−0.38, 10.32)	0.068
**Analgesic modality**^a^				
TAP	−1.60 (−2.36, −0.84)	**< 0.001**	−7.32 (−12.56, −2.08)	**0.006**
EOIP	−0.55 (−1.55, 0.45)	0.282	1.22 (−5.69, 8.13)	0.728
**Intraoperative MME**	0.00 (−0.01, 0.02)	0.827	−0.01 (−0.11, 0.08)	0.782

Data presented as β coefficients with 95 % CI from multiple regression analysis.

a Analgesic modality comparisons are made with SC as the reference group. BMI, Body Mass Index; CI, Confidence Interval; EOIP, External Oblique Intercostal Plane; MME, Morphine Milligram Equivalent; NRS, Numerical Rating Scale; SC, Standard Care; TAP, Transversus Abdominis Plane.

Further subgroup analysis accounting for block timing revealed that the analgesic benefit of TAP block was most pronounced when administered before surgical incision (Table 4). Pre-incision TAP block was associated with the largest reduction in both pain scores (β = −2.21, 95 % CI: −3.38 to −1.05, *p* < 0.001) and opioid consumption (β = −13.56, 95 % CI: −21.59 to −5.52, *p* = 0.001) compared to SC. While post-incision TAP block also reduced pain scores significantly (β = −1.32, 95 % CI: −2.18 to −0.47, *p* = 0.003), its effect on opioid consumption was not statistically significant (β = −4.55, 95 % CI: −10.44 to 1.35, *p* = 0.130). Neither pre- nor post-incision EOIP block showed significant differences from SC in pain scores or opioid requirements.

**Table 4:** Multivariable linear regression analysis of factors associated with pain scores and opioid consumption during the first eight postoperative hours, accounting for block timing.

	NRS 0h – 8h		MME 0h – 8h	
	β coefficient (95 % CI)	*p*-value	β coefficient (95 % CI)	*p*-value
**Age, years**	0.00 (−0.03, 0.03)	0.948	−0.12 (−0.30, 0.06)	0.186
**Male sex**	−0.06 (−0.81, 0.69)	0.875	−0.17 (−5.29, 4.95)	0.948
**BMI, kg.m^-2^**	−0.05 (−0.13, 0.02)	0.130	−0.38 (−0.87, 0.11)	0.131
**Diabetes mellitus**	−0.41 (−1.25, 0.42)	0.332	−1.86 (−7.60, 3.87)	0.522
**Living donor**	0.90 (0.12, 1.67)	**0.023**	4.91 (−0.42, 10.23)	0.071
**Block timing**^a^				
TAP pre-incision	−2.21 (−3.38, −1.05)	**< 0.001**	−13.56 (−21.59, −5.52)	**0.001**
TAP post-incision	−1.32 (−2.18, −0.47)	**0.003**	−4.55 (−10.44, 1.35)	0.130
EOIP pre-incision	−0.96 (−3.11, 1.20)	0.382	−2.14 (−16.95, 12.68)	0.777
EOIP post-incision	−0.45 (−1.54, 0.63)	0.409	1.99 (−5.45, 9.44)	0.598
**Intraoperative MME**	0.00 (−0.01, 0.02)	0.856	−0.02 (−0.11, 0.08)	0.748

Data presented as β coefficients with 95 % CI from multiple regression analysis.

a Block timing comparisons are made with SC as the reference group. BMI, Body Mass Index; CI, Confidence Interval; EOIP, External Oblique Intercostal Plane; MME, Morphine Milligram Equivalent; NRS, Numerical Rating Scale; SC, Standard Care; TAP, Transversus Abdominis Plane.

Postoperative complications were rare and occurred at similar rates across groups (Table 2). Overall, only 6 (2.5 %) patients were admitted to the ICU, 7 (3.0 %) patients developed a surgical site infection, and 5 (2.1 %) patients required reoperation. Median length of stay in PACU (3.3 [2.7, 4.1] hours) and hospital (6.5 [6.3, 8.5] days) were also comparable across groups.

## Discussion

In this retrospective study comparing different analgesic approaches for RT recipients, we found that TAP block, particularly when administered pre-incision, was associated with superior early postoperative pain control compared to SC. This benefit was evident in both reduced pain scores and decreased opioid consumption during the first eight postoperative hours. EOIP block did not demonstrate significant advantages over SC and was associated with higher opioid consumption compared to TAP block. However, these findings should be interpreted cautiously given the small EOIP sample size (*n* = 35). Notably, beyond eight hours, pain scores and opioid requirements were minimal across all groups. An unexpected finding was that living donor recipients experienced significantly higher pain scores regardless of the analgesic technique employed.

Our findings regarding TAP block efficacy align with previous evidence in RT literature. While the observed reduction in pain score with the TAP block was statistically significant, its clinical relevance warrants further consideration. With our institutional threshold for breakthrough pain defined as NRS > 5, the reduction from median NRS 7.0 (SC) to 5.0 (TAP) represents a clinically meaningful change that reduces the need for rescue analgesia. This clinical significance is further supported by the corresponding reduction in opioid consumption observed in the TAP group. A meta-analysis by Singh et al., which evaluated TAP blocks across ten trials (258 control patients and 237 receiving TAP blocks), demonstrated that TAP blocks reduced 24 hour postoperative opioid consumption by approximately 42.7 % in RT recipients and decreased intraoperative opioid requirements and pain scores in both early and delayed postoperative phases.^16^ However, the optimal timing of TAP block administration, whether pre- or post-incision, remains a topic of debate. While some studies suggest that pre-incision administration may offer superior analgesia,^17^^,^^18^ a recent meta-analysis reported that post-incision TAP blocks may be slightly more effective in reducing 24-hour postoperative opioid consumption and postoperative nausea and vomiting compared to pre-incision blocks.^19^ In our cohort, pre-incision TAP block was associated with the largest reductions in both pain scores and opioid consumption compared to SC, while post-incision administration was associated with more modest benefits for pain scores, without significantly affecting opioid consumption. These findings suggest a potential association between pre-incision TAP blocks and improved analgesic outcomes, possibly related to pre-emptive analgesia and preservation of anatomical plane integrity before surgical manipulation.

This is the first study to evaluate the efficacy of the EOIP block in RT recipients. We investigated the EOIP block based on literature precedent and its practical advantages (technical simplicity, distance from the surgical site), despite acknowledging its partial dermatomal coverage. Given the absence of consensus guidelines for RT analgesia, this prompted our systematic comparison. The EOIP block has demonstrated effectiveness in reducing pain scores and opioid requirements across various upper abdominal surgeries, including subxiphoid video-assisted thoracoscopic surgery,^20^ laparoscopic cholecystectomy,^21^, ^22^, ^23^ laparoscopic sleeve gastrectomy,^24^^,^^25^ living kidney donor open nephrectomy,^26^ and management of chronic post-surgical neuropathic pain.^27^ Despite its proven efficacy in these procedures, limited data exists regarding its application in RT.^13^ In our study, the EOIP block did not show a significant advantage over SC in RT recipients and was associated with higher opioid consumption compared to the TAP block. However, with only 35 patients in the EOIP group (including only six pre-incision blocks), our study was underpowered to detect potential benefits of this technique. Several factors may explain these findings. First, the dermatomal coverage of the EOIP block (T6‒T11)^13^ only partially overlaps with the Gibson incision (T10/11–L1/2), which is the standard surgical approach for RT at the study institution. This partial overlap may leave the lower segments of the surgical field inadequately covered. Second, as evidenced by our TAP block findings, the timing of block administration appears to be associated with different outcomes, with pre-incision blocks showing associations with superior efficacy.

Our study demonstrated minimal pain and opioid consumption beyond eight hours postoperatively, reinforcing the utility of multimodal analgesia and corroborating findings from other studies on this topic.^28^, ^29^, ^30^

Another interesting finding was that living donor transplantation was independently associated with significantly higher pain scores. This finding appears counterintuitive, given that living donor transplantation typically allows for more controlled operative conditions and shorter cold ischemia times than deceased donor procedures. Living donor recipients are generally healthier and younger, which might contribute to differences in pain perception compared to deceased donor recipients, who often have longer histories of dialysis and more comorbidities. However, this finding requires further investigation to understand the underlying mechanisms.

### Limitations and future direction

There are several limitations to our study. The retrospective nature of our study introduces potential selection bias, as the choice of analgesic technique was not randomized but based on individual anesthesiologist preference and expertise. The study was also limited by sample size disparities between groups, particularly in the EOIP group (*n* = 35), with notably few pre-incision blocks (*n* = 6). This imbalance might have affected our ability to detect the potential benefits of EOIP block, especially regarding the timing-dependent effects we observed with TAP blocks. The single-center design may affect the generalizability of our findings, as our institutional protocols and surgical approaches might differ from other transplant centers. Furthermore, due to the retrospective design, we could not assess several relevant outcomes, such as patient satisfaction, chronic pain development, or long-term functional recovery. Future prospective randomized trials should focus on comparing different regional analgesic techniques, particularly block timing. The EOIP block findings warrant further investigation with larger sample sizes. The observed association between donor type and postoperative pain also merits a dedicated study to understand the underlying mechanisms better and optimize pain management strategies.

## Conclusion

This retrospective study demonstrates that the TAP block, particularly when administered pre-incision, was associated with superior early postoperative pain control in RT recipients compared to SC. EOIP block showed no significant benefit, though the small sample size limits definitive conclusions. The critical period for pain management was the first eight postoperative hours.

## Data availability

The datasets generated and/or analyzed during the current study are available from the corresponding author upon reasonable request.

## Declaration of competing interest

The authors declare no conflicts of interest.
